# High Level Production of Monoclonal Antibodies Using an Optimized Plant Expression System

**DOI:** 10.3389/fbioe.2019.00472

**Published:** 2020-01-17

**Authors:** Andrew G. Diamos, Joseph G. L. Hunter, Mary D. Pardhe, Sun H. Rosenthal, Haiyan Sun, Bonnie C. Foster, Michelle P. DiPalma, Qiang Chen, Hugh S. Mason

**Affiliations:** ^1^Center for Immunotherapy, Vaccines and Virotherapy, The Biodesign Institute, Arizona State University, Tempe, AZ, United States; ^2^School of Life Sciences, Arizona State University, Tempe, AZ, United States

**Keywords:** plant-based biopharmaceuticals, pharming, transient expression, glycosylation, monoclonal antibodies, Zika virus, transient expression, heteromultimeric proteins

## Abstract

Biopharmaceuticals are a large and fast-growing sector of the total pharmaceutical market with antibody-based therapeutics accounting for over 100 billion USD in sales yearly. Mammalian cells are traditionally used for monoclonal antibody production, however plant-based expression systems have significant advantages. In this work, we showcase recent advances made in plant transient expression systems using optimized geminiviral vectors that can efficiently produce heteromultimeric proteins. Two, three, or four fluorescent proteins were coexpressed simultaneously, reaching high yields of 3–5 g/kg leaf fresh weight or ~50% total soluble protein. As a proof-of-concept for this system, various antibodies were produced using the optimized vectors with special focus given to the creation and production of a chimeric broadly neutralizing anti-flavivirus antibody. The variable regions of this murine antibody, 2A10G6, were codon optimized and fused to a human IgG1. Analysis of the chimeric antibody showed that it was efficiently expressed in plants at 1.5 g of antibody/kilogram of leaf tissue, can be purified to near homogeneity by a simple one-step purification process, retains its ability to recognize the Zika virus envelope protein, and potently neutralizes Zika virus. Two other monoclonal antibodies were produced at similar levels (1.2–1.4 g/kg). This technology will be a versatile tool for the production of a wide spectrum of pharmaceutical multi-protein complexes in a fast, powerful, and cost-effective way.

## Highlights

- Non-competing viral vectors allow simultaneous co-expression of 4 proteins
- Optimized plant expression vectors yield milligram quantities of mAb from a single plant leaf
- Humanized 2A10G6 mAb produced in plants retains its binding capacity and neutralization potency.

## Introduction

Antibody-based therapeutics are the largest sector of the global biopharmaceutical market, with sales exceeding 100 billion USD worldwide and sales predicted to reach 137–200 billion USD by 2022 (Grilo and Mantalaris, 2019). While most biopharmaceuticals have traditionally been produced in mammalian cell culture systems, plant-based recombinant expression systems have demonstrated significant advantages. Like mammalian systems, plants can carry out complex post-translational modifications necessary for the function of many biopharmaceuticals (Chen and Davis, 2016). However, unlike mammalian systems, which require large investment and operating costs (Ecker et al., 2015), plant systems do not require expensive cell-culture facilities and bioreactors (Buyel and Fischer, 2012). Furthermore, plant systems do not need to be grown in sterile conditions and, as plants lack animal pathogens, plant-based biotechnology has improved intrinsic safety over mammalian expression systems (Sack et al., 2015b). These factors allow highly scalable production of biopharmaceutical proteins with substantially reduced costs (Tusé et al., 2014; Walwyn et al., 2015; Nandi et al., 2016; Alam et al., 2018; Mir-Artigues et al., 2019). The cost-effectiveness of plant-based systems may especially benefit developing countries (Ma et al., 2013).

In addition, advances in plant engineering have resulted in the ability to produce tailor-made glycans. The glycosylation state of the antibody is crucial for its stability and function (Mastrangeli et al., 2019). In comparison to mammalian cells which have highly heterogeneous glycoforms that may be detrimental for biopharmaceutical production, advances in plant glycoengineering have allowed the production of monoclonal antibodies (mAbs) with more homogenous human-like glycans (Montero-Morales and Steinkellner, 2018). By removing the endogenous plant-specific β1,2-linked xylose and α1,3-linked fucose, a variety of plant-made antibodies have demonstrated improved immune receptor binding and greater potency compared to commercially available antibodies produced in mammalian cells (Zeitlin et al., 2011; Marusic et al., 2018). Remarkably, the entire human sialyation pathway has been transferred into plants (Castilho et al., 2010, 2012). These advances in glycoengineering have been used in several practical applications. Antibodies made in glycoengineered plants have been successfully used to treat Ebola virus disease in rhesus macaques and humans (Olinger et al., 2012; Lyon et al., 2014; Qiu et al., 2014) and the first in-human clinical trials have been carried out using plant-made antibodies (Ma et al., 2015). High expressing, safe, and efficacious plant-made antibody therapies have also been developed for dengue virus (Dent et al., 2016), West Nile virus (Sun et al., 2018), and chikungunya virus (Hurtado et al., 2019), while a variety of antibody-based immune complex vaccines have been developed in plants and shown promising efficacy in mouse immunization trials (Chargelegue et al., 2005; Pepponi et al., 2014; Kim et al., 2015; Mason, 2016; Webster et al., 2018; Diamos et al., 2019b).

Plant-based biopharmaceuticals can be made in plant cells, tissues, or whole plants that are either stably transformed with the target gene or expressed transiently via agroinfiltration (Buyel, 2019). Since the viability of plant-based expression systems depends strongly on the yield of the target product (Fischer et al., 2015; Sack et al., 2015a), efforts to increase the expression of a desired protein have focused on rigorously optimizing every step in the lifecycle of the target gene product, from delivery of the transgene to the plant cell to the purification of the fully assembled protein. We have developed a plant expression system based on the geminivirus bean yellow dwarf virus (BeYDV), which replicates the target gene to a high copy number in the plant nucleus (Huang et al., 2009, 2010). By optimizing transgene codons and removing deleterious sequences (Geyer et al., 2007, 2010; Mathew et al., 2011), improving delivery of the transgene by *Agrobacterium* (Diamos et al., 2016), modifying vector replication (Diamos and Mason, 2018c), and improving downstream transcription and translational processes (Diamos and Mason, 2018a; Rosenthal et al., 2018), this system is capable of producing a variety of biopharmaceutical proteins at yields equal to or greater than the highest levels reported in plant-based systems. In addition, this streamlined system requires only 4–5 days from the delivery of the transgene to the harvesting of plant material that contains the desired protein (Diamos and Mason, 2018b; Hunter et al., 2018). The optimized BeYDV vectors also offer advantages over expression systems based on RNA viruses. While RNA-based systems need to use multiple non-competing viruses to express separate proteins in the same cell, BeYDV vectors are non-competing and can be used to produce heteromultimeric proteins from a single vector (Huang et al., 2010). Finally, the large host range of BeYDV allows high level protein production in a variety of dicot plants (Diamos and Mason, 2018a).

In the present study, we show that optimized plant expression BeYDV vectors can simultaneously coexpress high levels of two, three, or four fluorescent proteins in a non-competitive manner, leading to near equal expression of each protein. Using these optimized vectors, milligram quantities of three different mAbs can be produced from a single plant leaf. We also demonstrate the ability to produce a chimeric antibody against a highly conserved fusion loop epitope found on flaviviruses and show that the antibody is correctly assembled in plants, can be purified to near-homogeneity with a simple purification procedure, and retains the binding ability of the original murine antibody.

## Materials and Methods

### Expression Vector Construction

The construction of plasmids pBYGFP and pBYDsRed has been previously described (Huang et al., 2010). For the construction of pBYCFP, the CFP gene (Accession number EU530627) was PCR amplified from plasmid pIBT-PR7:eCFP (a kind gift from Dr. Z. Huang) with primers GFP-BsaF and GFP-PacI (Table 1), digested with BsaI and PacI, and ligated with pBYR7, a derivative of pBYR2 (Chen et al., 2011). The CFP cassette was obtained by PCR with primers U35S-SpeF and Ext6-SalR using pBYCFP as a template, digested with SpeI-SalI, and ligated with pBY-GR digested SpeI-SalI, to yield the single-replicon three-expression cassette vector pBY-GCR. An optimized BeYDV expression vector for GFP was created by three fragment ligation: the backbone vector pBYR2e-MRtxGM (Diamos et al., 2016) was obtained by XhoI-KpnI digestion; the Rb7 MAR was also obtained from pBYR2e-MRtxGM by KpnI-EcoRI digestion; a fragment containing the PsaK2 5′ UTR, GFP, tobacco extension intronless 3′ UTR, and NbACT 3′ UTR was obtained from pPS-OGFPM-EA (Diamos and Mason, 2018a) by XhoI-EcoRI digestion. The resulting vector, pBYKEAM-GFP, was used to create individual expression vectors for DsRed, CFP, and YFP by a three fragment ligation: the backbone from pBYKEAM-GFP was obtained by XhoI-AgeI digestion; the Rep/MAR genes were obtained by AgeI-SacI digestion; and DsRed/CFP/YFP were obtained by XhoI-SacI digestion of pBYDsRed/pBYCFP/pBYYFP (gifts from Z. Huang, Arizona State University).

**Table 1:** Oligonucleotides used in this study.

**Primer**	**Sequence**
GR5-1	GCGGTACCCAATTCGCCCTATAGTGAGTCG
GR5-2	GTGTCGTGCTCCACCATGCCGTCGACGCACTAGTCGATA GCTTGATGCATGTTGTC
GR3-1	GACAACATGCATCAAGCTATCGACTAGTGCGTCGACGGC ATGGTGGAGCACGACAC
GR3-2	GAGAGCTCCACCGCGGTGGC
U35S-Spe-F	GGACTAGTGACCCTCCTGCAGGTCAAC
Ext3-Sal-R	GCGTCGACCGAAACTGAACAAAACATACAC
ZprM-Bsa-F	ggGGTCTCTCgTGGTGCCGAGGTCACTAGAC
M13-RHT	GGAAACAGCTATGACCATG
35S-F	AATCCCACTATCCTTCGC
BASP-G-BSA-R	gcGGTCTCCACCAGAAGCAAGAGAAGC
GFP-Bsa-F	aggGGTCTCgTGGTatggtgagcaagggcga
GFP-PacI	gcgttaattaaaccaccatggtgagcaagggcgaggagc
Ext6-SalR	GCg tcg acC GAA ACT GAA CAA AAC ATA CAC
GR5-1	GCG GTA CCC AAT TCG CCC TAT AGT GAG TCG
GR3-1	GAC AAC ATG CAT CAA GCT ATC GAC TAG TGC GTC GAC GGC ATG GTG GAG CAC GAC AC
GR3-2	GAG AGC TCC ACC GCG GTG GC
6D8-CL-F	CCATCTGTCTTCATCTTtCCt
Ext3i-R	CAATTTGCTTTGCATTCTTGAC

#### BeYDV-Based Tandem Dual Replicon Constructs

pBYGFPDsRed.R was described previously (Huang et al., 2010) and renamed as pBY-G(SL)R in this study. Construct pBY-GR was designed to contain two expression cassettes in tandem and to be flanked by LIR and SIR. For the construction of pBY-GR. Primer sets GR5-1/GR5-2 9 and GR3-1/GR3-2 were used for initial amplification in separate PCR reactions using pBY-G(SL)R as a template. The resulting PCR fragments were mixed and amplified using primers GR5-1 and GR3-2, complementary to the ends of the two initial fragments. The resulting PCR product was digested with SacI-KpnI and ligated with pBY-G(SL)R digested with SacI-KpnI, to yield pBY-GR.

#### Dual Cassette Single Replicon Vectors

**Figure 1:**
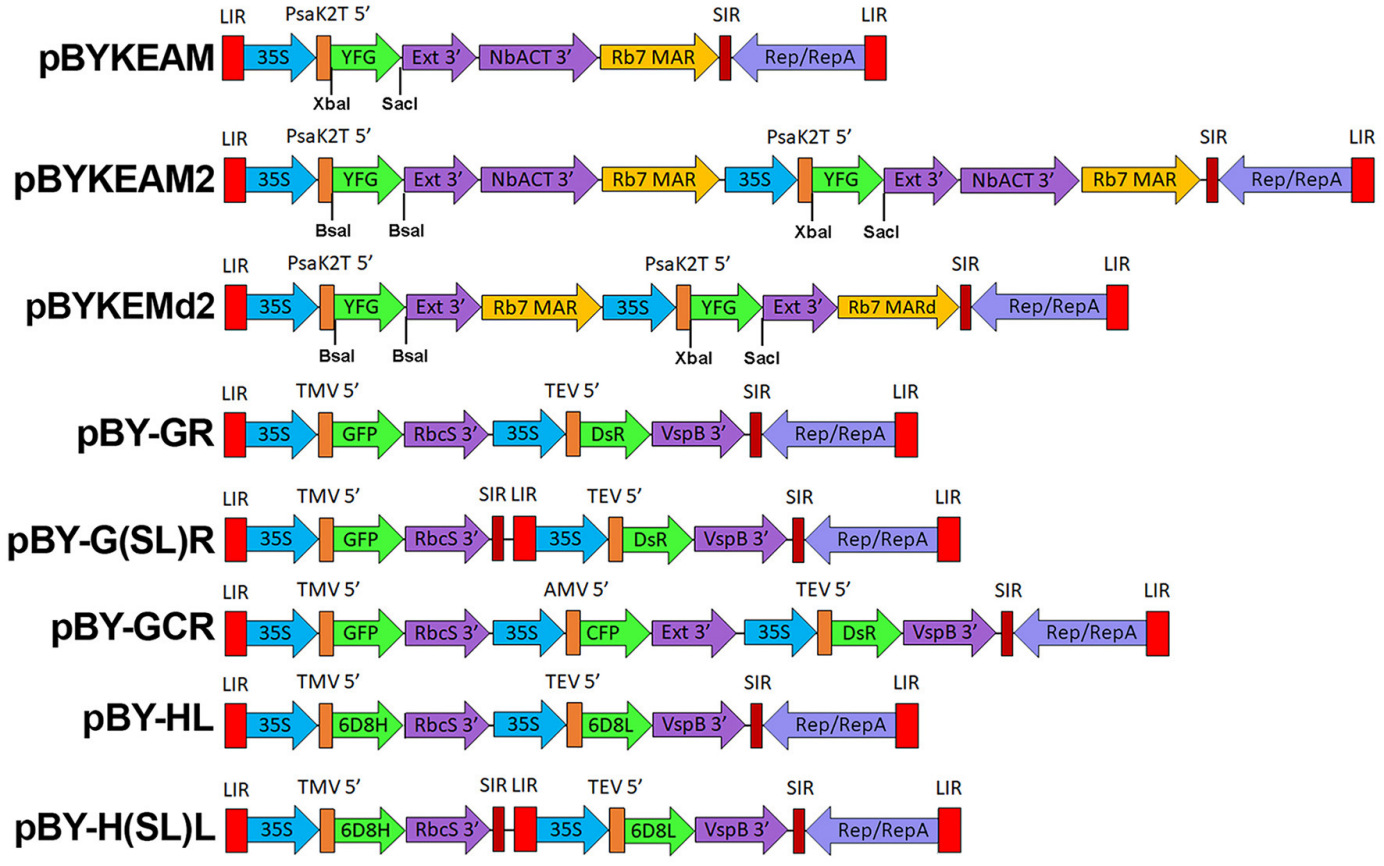
A generalized schematic of the plant expression vectors used in this study. pBYKEAM is a replicating plant expression vector based on BeYDV with optimized 5′ and 3′ UTRs and is the vector of choice for high expression of a single target gene (Diamos et al., 2016; Diamos and Mason, 2018a; Rosenthal et al., 2018). pBYKEAM2 allows simultaneous co-expression of two genes from a single replicon containing optimized 5′ and 3′ UTRs. pBYKEMd2 is a previous iteration of pBYKEAM2 that does not contain double terminators. The remaining vectors contain multiple expression cassettes arranged as either a single large replicon or multiple replicons (when the gene cassettes are separated by SIR/LIR) with unoptimized 5′ and 3′ UTRs. For expression of antibodies, the barley alpha amylase signal sequence for ER-targeting is also present at the start of the gene. LIR, the long intergenic region from BeYDV; 35S, the 35S promoter with duplicated enhancer region from cauliflower mosaic virus; PsaK2T 5′ , the truncated 5′ UTR from the *N. benthamiana* psaK gene; Ext 3′ , the tobacco extensin terminator with intron removed; NbACT 3′ , the 3′ UTR from the *N. benthamiana* ACT3 gene; Rb7 MAR, the tobacco Rb7 matrix attachment region; Rb7 MARd, a truncation of the Rb7 MAR to remove unwanted restriction enzyme sites; SIR, the short intergenic region from BeYDV; Rep/RepA, the replication proteins from BeYDV; TMV 5′ , the 5′ UTR from tobacco mosaic virus; RbcS 3′ , the 3′ UTR from the pea rbcS gene; VspB 3′ , the 3′ UTR from the soybean vspB gene; AMV 5′ , the 5′ UTR from alfalfa mosaic virus; TEV 5′ , the 5′ UTR from tobacco etch virus; YFG, insertion site for the gene of interest; GFP, green fluorescent protein; DsR, DsRed fluorescent protein; YFP, yellow fluorescent protein; CFP, cyan fluorescent protein; 6D8H, the 6D8 heavy chain; 6D8L, the 6D8 light chain.

pBYKEMd2 (Figure 1) is based upon pBYR11eMa-h6D8-L2 (Diamos et al., 2019b) and contains the optimized PsaK2 5′ UTR, tobacco extensin terminator, and Rb7 MAR (Diamos et al., 2016). The first cassette contains BsaI sites in inverted orientations in order to permit insertion of a coding sequence between PsaK2 5′ UTR and Ext 3′, and the second cassette enables insertion of a coding sequence between unique XbaI and SacI sites (Figure 1). The insertion of the NbACT 3′ UTR (Diamos and Mason, 2018a) downstream of the Ext 3′ in pBYKEMd2 produced pBYKEAM2, having double terminator cassettes.

#### mAb 2A10G6 and Zika Antigen Cloning

The amino acid sequences of the murine heavy and light chain variable regions (VH and VL) of anti-Zika virus mAb 2A10G6 were obtained from the NCBI Protein Databank (5JHL_H and 5JHL_L). The VH and VL amino acid sequences were codon-optimized for expression in *N. benthamiana* plants (Geyer et al., 2010), and the nucleotide sequences were synthesized as gBlocks® Gene Fragments (Integrated DNA Technologies, idtdna.com). PCR of the plasmid pBYR9-K3 was conducted with primers 6D8 CL-F and Ext3i-R to copy the human light chain constant region (CL) from humanized mAb 6D8 anti-Ebola antibody (Huang et al., 2010). The VL and CL fragments were fused by overlapping PCR, and the resulting fragment was digested with XbaI and SacI for insertion into pBYKEMd2 (Figure 1), to create pBYKEMd2-5JHL-K. The murine VH gene block was digested with XhoI and NheI and fused with the humanized heavy chain human CH region (Huang et al., 2010). The dual cassette vector containing both H and L chains was created by insertion of the chimeric VH-CH segment into the BsaI sites of pBYKEMd2-5JHL-K.

Zika virus antigens were produced to test mAb binding. The coding DNA sequence of ZIKV structural proteins (GenBank accession AMC13911) was synthesized with codons optimized for *N. benthamiana* (idtdna.com). Zika soluble ectodomain (amino acids 1-403), here called ZsE, fused via the C-terminus to a 6H tag, was created by ligating two DNA fragments: (1) pBYe3R2K2Mc- L2(14-122) (Diamos et al., 2019b) was digested XhoI-SpeI; (2) the Zike E soluble ectodomain was digested XhoI-SpeI. The resulting vector is pBYe3R2K2Mc-BAZsE6H. ZIKV prM, M, and E protein (prME) was obtained by PCR on template pUC57-ZCME-F (a kind gift from Lydia Meador) with primers ZprMBsaF and M13RHT, creating BsaI and SacI sites. To amplify a segment containing the barley alpha amylase signal peptide (BASP), PCR was performed on pBYR2eK2M-BAZE with primers 35S-F and BASP-G-Bsa-R. The purified PCR products for ZprME and BASP were digested with BsaI-SacI and BsaI-XhoI, respectively. The backbone vector pBYR2eK2MC-GFP (Diamos and Mason, 2018c) was digested with SacI-XhoI and ligated with the PCR products to make pBYR2e3K2M-BAZprME (referred to in the text as ZprME).

#### mAb HSV8 Cloning

The anti-herpes simplex virus human mAb HSV8 H and L chain coding sequences were a kind gift of Larry Zeitlin (MAPP Biopharmaceutical, San Diego, CA). The VL and VH coding segments were fused to the mAb 6D8 CL and CH coding segments, respectively, as described above for mAb 2A10G6. The L sequence was inserted into pBYKEMd2 (Figure 1) via XbaI-SacI, followed by H sequence insertion via BsaI-BsaI, to produce pBYKEMd2-HSV8.

### Protein Production, Extraction, and Purification

*Agrobacterium tumefaciens* strain EHA105 was transfected with expression vectors via electroporation. Resulting strains were confirmed using PCR and restriction digestion of purified plasmids. Confirmed *Agrobacterium* strains were grown overnight at ~30°C in YENB media + 50 mg/L kanamycin and 2.5 mg/L rifampicin. *Agrobacterium* was pelleted for 10 min at 5,000 g. The pellets were resuspended in infiltration buffer [10 mM 2-(N-morpholino) ethanesulfonic acid (MES), pH 5.5 and 10 mM MgSO4) to a final OD600 of 0.2 for single vector infiltrations. For multi-vector infiltration experiments, *Agrobacterium* were mixed such that the final OD_600_ of each vector was equal to 0.2. *Agrobacterium* suspensions were infiltrated using a syringe without needle into the leaves of 5–6 week old glycoengineered *N. benthamiana* silenced for production of the plant-specific β1,2-linked xylose and α1,3-linked (Castilho and Steinkellner, 2012). Infiltrated leaves were harvested 4 days post infiltrations (DPI) unless otherwise noted.

0.1-g samples of leaf tissue expressing fluorescent protein were homogenized in 1:5 w/v extraction buffer (25 mM Tris-HCL, 125 mM NaCl, 3 mM EDTA, pH 8.0 with 50 mM sodium ascorbate, and 2 mM phenylmethylsulfonyl fluoride (PMSF) added before extraction). The homogenization process was conducted as follows: 12–14 ZnO beads, 2.0 mm (Fisher Scientific, Waltham, MA, United States), were added to tubes containing leaf samples and extraction buffer. The tubes were bead beaten using a Bullet Blender machine (Next Advance, Averill Park, NY, United States) for two 5-min rounds. Homogenized leaf tissue was then rotated at room temperature or at 4°C for 10–15 min. The samples were spun down at 13,000 g for 10–20 min at 4°C in a 5417R centrifuge (Eppendorf, Hauppauge, NY, United States) and the supernatant transferred to a new tube. The samples were recentrifuged at 13,000 g for 10–20 min at 4°C to obtain a clarified extract free of major plant contaminants.

For small-scale antibody experiments, 0.05-g leaf samples were collected at 5 DPI. The samples were then homogenized in ice-cold, 1:5 w/v extraction buffer at pH 8.02 (25 mM Tris-HCl, 125 mM NaCl, 3 mM EDTA, 0.1% Triton X-100, 50 mM sodium ascorbate, and 2 mM PMSF). Homogenized leaf extracts were rotated at 4°C for 18 min, then centrifuged at 13,000 g for 12 min. Following the centrifugation, the supernatants were transferred to new tubes for further analysis. An acid precipitation of the samples was conducted by adding 1N phosphoric acid so that the final acid volume was 4% of the soluble leaf extract (~pH 4.1). Following a brief centrifugation at 13,000 g for 2 min, the supernatant of the acid-precipitated samples were collected for analysis by SDS-PAGE and Western blot.

The purification of the c2A10G6 antibody was conducted as described in Diamos et al. (2019b). Following the purification, samples from the elutions were run on an SDS-PAGE gel to assess purity.

For the extraction of ZsE, 0.1 g leaf samples were harvested 5 DPI and homogenized in ice-cold 1:5 w/v extraction buffer at pH 8.02 (25 mM Tris-HCl, 125 mM NaCl, 3 mM EDTA, 0.1% Triton X-100, 50 mM sodium ascorbate, and 2 mM PMSF). Homogenized leaf extracts were rotated at 4°C for 12–15 min, then centrifuged at 13,000 g for 12 min. An uninfiltrated leaf sample was prepared using the same procedure. After centrifugation, the supernatants were collected for use in an ELISA.

Leaf tissue samples infiltrated with ZprME were harvested 4 DPI. Each leaf sample of 0.2 g was homogenized with a 1:4 ratio w/v of PBS, pH 7.4, supplemented with 1% Triton X-100, 0.1 M sodium ascorbate, and 1 mM PMSF. An uninfiltrated sample was prepared in the same manner. The homogenized material was then centrifuged at 10,000 g for 10 min at room temperature. After centrifugation, the supernatant was collected for further analysis.

### SDS-PAGE, Fluorescence Imaging, and Western Blot Analysis

Clarified extracts were mixed with SDS sample buffer (50 mM Tris-HCl, pH 6.8, 2% SDS, 10% glycerol, 0.02 % bromophenol blue) under either reducing (0.5 M DTT added) or non-reducing conditions (no DTT added). Reducing samples were boiled for 10 min. Samples mixed with non-reducing buffer were not boiled, but were incubated at room temperature for ~5 min after the addition of sample buffer. Samples were separated on 4–15% polyacrylamide gels (Bio-Rad, Hercules, CA) as well as stain-free 4–15% polyacrylamide gels (Bio-Rad, Hercules, CA). Florescent protein samples run under non-reducing conditions on standard 4–15% polyacrylamide gels were visualized on a UV-transilluminator and photographed for analysis. Florescent protein samples run under both reducing and non-reducing conditions on either standard or stain-free gels were analyzed using Coomassie stain (Bio-Rad, Hercules, CA, United States). A similar protocol was followed for the gels containing antibody samples. The silver stain analysis was conducted using a Pierce® Silver Stain Kit (Thermo Fisher Scientific, Waltham, MA, USA). The manufacturer's instructions were followed with a slight modification in that the gel was fixed with glutaraldehyde (12.5%) instead of 30% ethanol. All other steps were followed according to the manufacturer's protocol.

Antibody samples were electroblot transferred to PVDF membranes. PVDF membranes were blocked with 5% dry milk in PBST (PBS with 0.05% Tween-20) at 37°C for 1 h. Then, membranes were washed thrice with PBST before incubation with a 1:1000 dilution of goat anti-human IgG (kappa only) HRP conjugate (Southern Biotech, Birmingham, AL, USA). Bound antibody was detected with luminol reagent (Santa Cruz Biotechnologies, Santa Cruz, CA).

Samples of ZprME were electroblot transferred to PVDF membranes. PVDF membranes were blocked in 5% PBSTM overnight, washed with PBST, and probed with purified c2A10G6. After a 2-h room temperature incubation, the membrane was washed in PBST and probed with a 1:5,000 dilution of a HRP-conjugated goat anti-human IgG antibody (Southern Biotech, Birmingham, AL, USA). Bound antibody was detected with luminol reagent (Santa Cruz Biotechnologies, Santa Cruz, CA).

### Plant DNA Extraction and DNA Replicon Analysis

Total plant DNA was extracted using DNeasy Plant Mini kit (Qiagen) according to the manufacturer's instructions. Purified DNA (1 μg) was digested with the indicated restriction enzymes (**Figure 3**) and run on a 1% agarose gel with ethidium bromide for DNA staining.

### Analysis of Fluorescent Proteins

GFP and DsRed fluorescence intensity was examined on a microplate reader (Spectra Max M2, Molecular Device) at room temperature. TSP samples (25 μg) were loaded to black-wall 96-well plates (Corning) in duplicate and read with excitation and emission wavelength of 485 and 538 nm, respectively, for GFP, and 544 and 590 nm for DsRed. The fluorescence value of the negative control (extract of un-infiltrated plant leaf) was subtracted before graphing. Expression levels are reported as fluorescence units (FU) per 25 μg TSP (**Figure 3B**). Fluorescence microscope images were taken using a Zeiss LSM 5 DUO (Carl Zeiss) laser scanning confocal microscope. Infiltrated leaf tissue sections were mounted with water and imaged with a Zeiss EC Plan-Neofluar 40X/1.3 oil immersion lens. Fluorescence signals for GFP, CFP, and DsRed were sequentially scanned with excitation lasers of 488, 458, and 543 nm, respectively, and detection windows of 550–560, 470–500, and 614–646 nm, respectively. For plant chlorophyll autofluorescence detection, the excitation laser of 633 nm with detection window of 630–700 nm was used. All images were taken at 512 × 512 pixel resolution covering an area of 318 × 318 μm^2^. An 8-line average was applied to all scans with the scan speed set to 6.39 μs/pixel.

Fluorescent protein production was analyzed using ImageJ software. The band intensity of each individual protein (GFP, DsRed, CFP, and YFP) was measured and their values normalized using endogenous plant proteins to control for total protein loading.

### ELISA Quantification of Antibody Expression Levels

A 96 well high-binding polystyrene plate (Corning Inc, Corning, NY, USA) was coated with a 1:500 dilution of unlabeled goat anti-human IgG (Southern Biotech, Birmingham, AL, USA) and incubated at 37°C for 1 h. After being washed with PBST, the plates were blocked with 5% non-fat dry milk in PBST for 30 min and washed with PBST. Multiple dilutions, ranging from 1:600 to 1:4800, of clarified plant extracts containing the different antibodies were added to the plate. Previously quantified, plant-produced antibody that was purified by protein G column chromatography was included as a standard and positive control while samples of uninfiltrated crude extract were added for a negative control. After a 1-h incubation, the plate was washed three times in 1x PBST. Bound antibody was detected by incubating the plate with a 1:2000 dilution of goat anti-human IgG (kappa only) HRP conjugate (Southern Biotech, Birmingham, AL, USA) for 1 h at 37°C. The plate was then washed five times with PBST, developed with TMB substrate (Thermo Fisher Scientific, Waltham, MA, USA), and the absorbance read at 450 nm.

### ELISA Testing the Functional Characterization of cA10G6

Clarified plant extract containing the Zika soluble envelope (ZsE) was prepared as detailed above. An uninfiltrated leaf sample was also prepared in the same manner. Then, 50 μL of extract was bound to a 96 well high-binding polystyrene plate (Corning Inc, Corning, NY, USA). After a 1-h incubation at 37°C, the plate was blocked with 5% PBSTM for 20 min, washed 3 times with PBST, and incubated with purified c2A10G6. After a 1-h incubation at 37°C, the bound antibody was detected by incubating the plate with a 1:2000 dilution of goat anti-human IgG (kappa only) HRP conjugate (Southern Biotech, Birmingham, AL, USA). After an hour-long incubation, the plate was washed four times in PBST, developed with TMB substrate (Thermo Fisher Scientific, Waltham, MA, USA), and the absorbance read at 450 nm.

### ZIKV Neutralization and Plaque Assay

Plaque reduction neutralization test (PRNT) was performed based methods modified from previous publications (Lai et al., 2018; Yang et al., 2018). Specifically, mAb c2A10G6 and 6D8 (negative control mAb) were serially diluted in phosphate-buffered saline (PBS), while ZIKV (PRVABC59, ATCC# VR-1843) was diluted in serum-free DMEM to a concentration of 100 plaque forming units (PFU) per well. c2A10G6 and 6D8 were then mixed with ZIKV and incubated for 1 h at 37°C. ZIKV attachment to Vero cells (ATCC # CCL-81) was accomplished by adding the ZIKV-mAb mixture to Vero cells in a 12-well tissue culture plate (90–95% confluence) and incubated for 1 h at 37°C. Cells were then overlaid with fresh media (complete DMEM containing 1% agarose, Lonza) and incubated for 3 days at 37°C. Plaques were visualized by fixing cells with 4% paraformaldehyde (PFA, MilliporeSigma, MA) followed by staining with 0.2% crystal violet. Percent (%) neutralization was calculated as: [(number of plaque per well without mAb) – (number of plaque per well of diluted mAb)/(number of ZIKV plaque per well without mAb) x 100]. The half maximal effective concentration (EC_50_) of c2A10G6 mAb was calculated using GraphPad Prism software (Version 8.3).

## Results

### Simultaneous Co-expression of Two Fluorescent Proteins Using Optimized BeYDV Vectors

**Figure 2:**
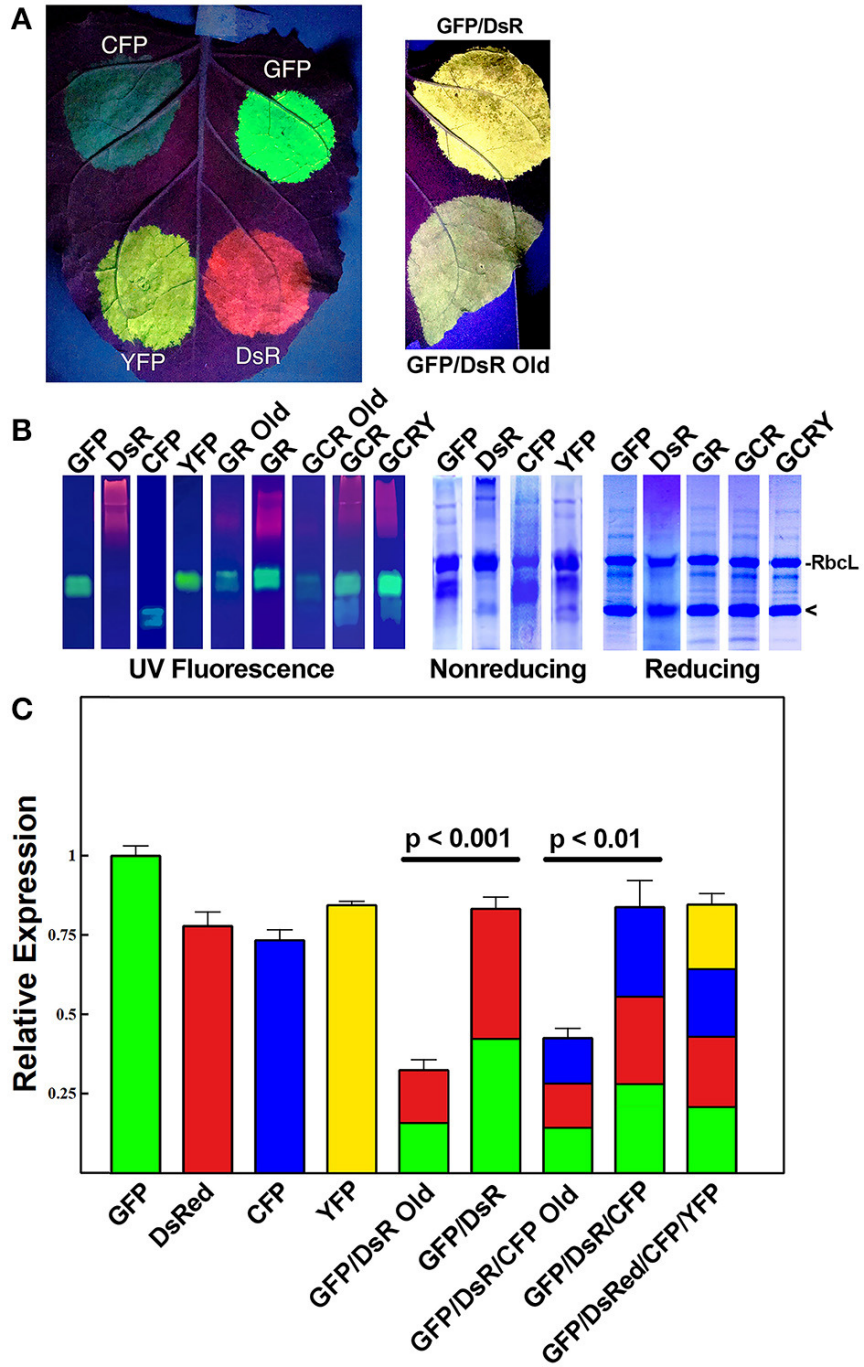
Simultaneous co-expression of up to four proteins with comparisons of optimized BeYDV vectors to unoptimized BeYDV vectors. Leaves of *N. benthamiana* were agroinfiltrated with either optimized (pBYKEAM or pBYKEAM2) vectors or unoptimized vectors (pBY-GR, pBY-GCR, referred to as “old”) for GFP, DsRed, CFP, and YFP in various combinations and **(A)** imaged at 5 dpi under UV illumination; or **(B)** separated by non-reducing or reducing SDS-PAGE and either viewed under UV transillumination or stained with Coomassie gel. Rubisco large subunit (RbcL) and the monomeric-sized band of all fluorescent proteins are indicated. **(C)** The relative total expression of each combination of constructs was analyzed using ImageJ software to quantify the band intensity of SDS-PAGE reducing gels. The mean band intensity is given ± standard error from 3 independently infiltrated leaf samples, where the expression of pBYKEAM-GFP was arbitrarily defined as 1. The bar colors are estimates of the relative production of each construct by non-reducing SDS-PAGE followed by ImageJ analysis using 3 independently infiltrated leaf samples. Statistical significance was calculated using student's *t*-test.

We previously reported that two proteins could be efficiently and simultaneously produced by either a single replicating BeYDV vector or by multiple codelivered vectors (Huang et al., 2010). In order to study the co-expression of multiple genes, we created an optimized vector by the following method: the coding sequences for GFP and DsRed were incorporated into vectors containing a single BeYDV replicon, the NbPsaK 5′ UTR, a double terminator consisting of the intronless tobacco extensin terminator fused to the NbACT3 3′ UTR, and the Rb7 matrix attachment region (Figure 1) (Diamos et al., 2016; Diamos and Mason, 2018a,c). These vectors were named either “pBYKEAM” (signifying that it contained one expression cassette) or “pBYKEAM2” (signifying that it contained two expression cassettes). The original vector without the optimized components (Huang et al., 2010) (referred to as pBY-GR in this study) was agroinfiltrated into the leaves of *N. benthamiana* along with optimized vectors expressing GFP or DsRed either alone or coinfiltrated together. While the unoptimized vector produced weak yellow fluorescence typical of GFP and DsRed expressed together, the optimized vector produced bright yellow fluorescence (Figure 2A). Protein extracts from agroinfiltrated leaf spots were analyzed by SDS-PAGE under UV light or after staining with Coomassie Brilliant Blue.

Under non-reducing conditions, the ~27 kDa GFP is typically visible as an approximately dimer-sized fluorescent band. In contrast, the ~26 kDa DsRed runs as a very diffuse high molecular weight fluorescent band (Figure 2B, compare lanes “GFP” and “DsRed”). The optimized vector produced notably increased levels of fluorescence of both GFP and DsRed compared to the old, unoptimized vector (Figure 2B, compare lanes “GR old” to “GR”). To more accurately assess the difference in expression, reducing SDS-PAGE conditions were used to collapse the higher molecular weight bands into a single monomeric-sized band. Quantifying the band intensity revealed that the optimized vector produced 2.54-fold (*p* < 0.001) more GFP and DsRed than the original vector. We estimate this yield at up to 50% of the plant total soluble protein or 3-5 g recombinant protein per kilogram leaf fresh weight (g/kg LFW) (Figure 2B, reducing lanes).

Although the fluorescence intensity of each protein may not correlate directly with yield due to differences in their peak fluorescence, we were able to compare the relative fluorescence of the same protein expressed either alone or coexpressed. Using samples taken from constructs GFP, DsRed, and GR each agroinfiltrated nearby on the same leaves to reduce variability, we compared the band intensity of GFP expressed by itself to the GFP band intensity in construct GR, revealing a 52% ± 4 decrease in GFP fluorescence. Similar results were found when comparing DsRed alone to construct GR. Therefore, our data suggests construct “GR” produces roughly half as much GFP and DsRed as each construct expressed alone. Since the total yield of recombinant protein is nearly equal between either GFP or DsRed alone and construct GR (Figure 2B, reducing lanes, compare “GFP” and “DsR” to “GR”), these findings indicate that roughly equal levels of GFP and DsRed are made in construct GR (Figure 2C, colored to indicate the relative expression of each construct).

**Figure 3:**
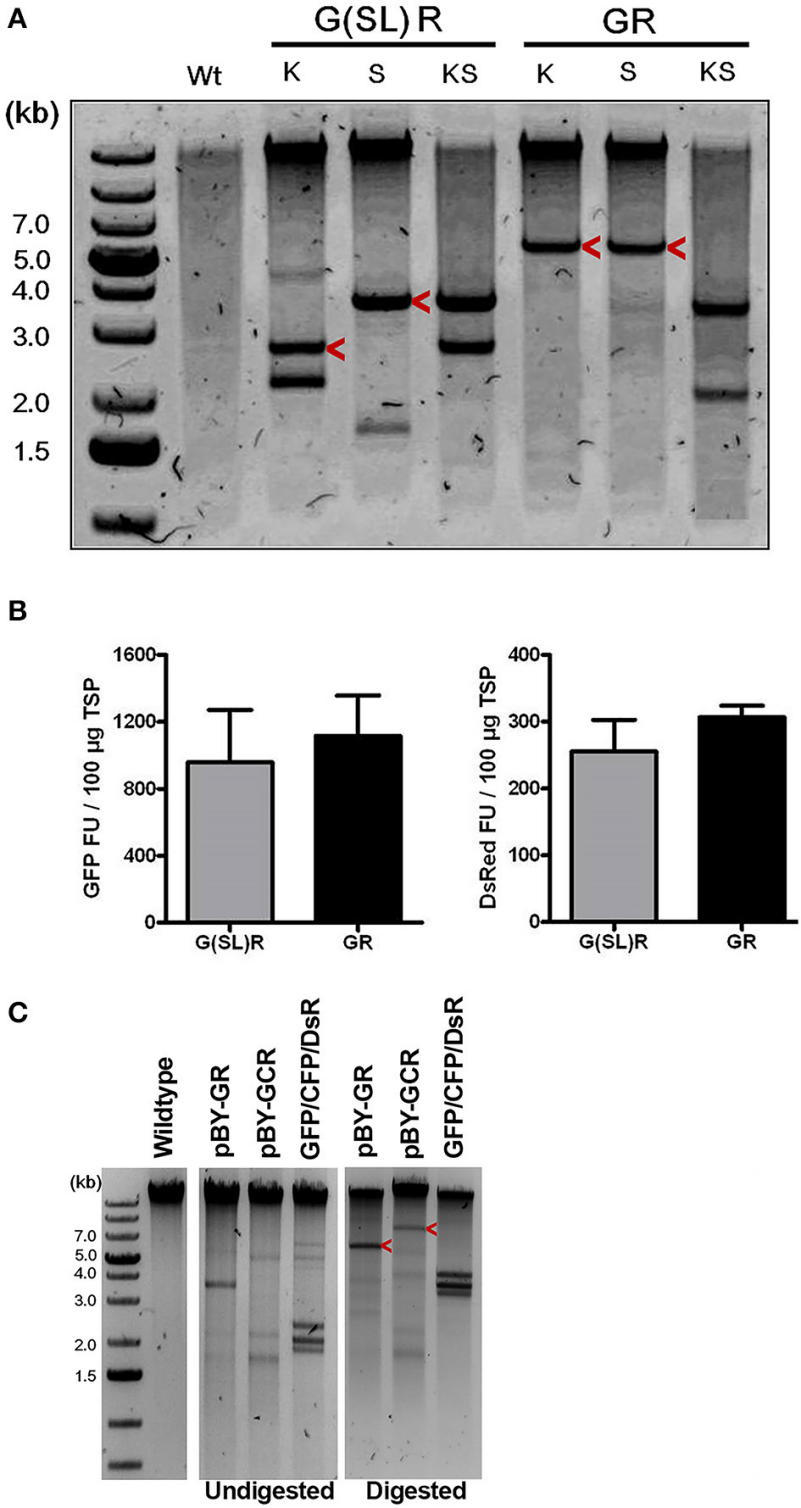
The role of replicon size and configuration in expression and replication of BeYDV vectors. **(A)** Leaf DNA was extracted at 3 DPI from uninfiltrated (Wt), or samples infiltrated with the indicated vectors, then digested with indicated restriction enzymes (K, KpnI; S, SacI) and run on a 1% agarose gel. G(SL)R refers to pBY-G(SL)R; GR refers to pBY-GR. Replicon positions are indicated with arrow head. **(B)** Fluorimetric analysis of GFP and DsRed accumulation showing efficient co-expression of two fluorescent proteins from either single [pBY-GR] or dual [pBY-G(SL)R] replicon vectors. Dilutions of total soluble protein extracts were subjected to spectrofluorimetry using excitation and emission wavelengths of 485 and 538 nm for GFP, and 544 and 590 nm for DsRed. G(SL)R, pBY-G(SL)R; GR, pBY-G(SL)R. Data are means ± S.D. from three independently infiltrated samples. **(C)** DNA from leaves of uninfiltrated (Wt), or infiltrated with indicated vectors were separated on 0.8% agarose gels before and after restriction enzyme digestion. GFP+CFP+DsRed indicates co-infiltrated sample with *Agrobacterium* mixture of pBY-GFP, pBY-CFP, and pBY-DsRed. Restriction enzyme XhoI was used for pBY-GR and GFP+CFP+DsRed. For pBY-GCR, restriction enzyme SalI was used. Expected replicon positions are indicated with arrow heads.

Since BeYDV replicons can be arranged either as individual tandem replicons that are separately released and replicated, or as a single large replicon containing multiple expression cassettes, we tested whether one configuration of viral replicons was more optimal than the other for expression of multiple proteins. A single replicon containing GFP and DsRed expression cassettes was compared to a vector containing GFP and DsRed in separate replicons. In either case, no significant differences in expression were observed, and both configurations were efficiently replicated (Figures 3A,B).

### Simultaneous Co-expression of Up to Four Fluorescent Proteins

**Figure 4:**
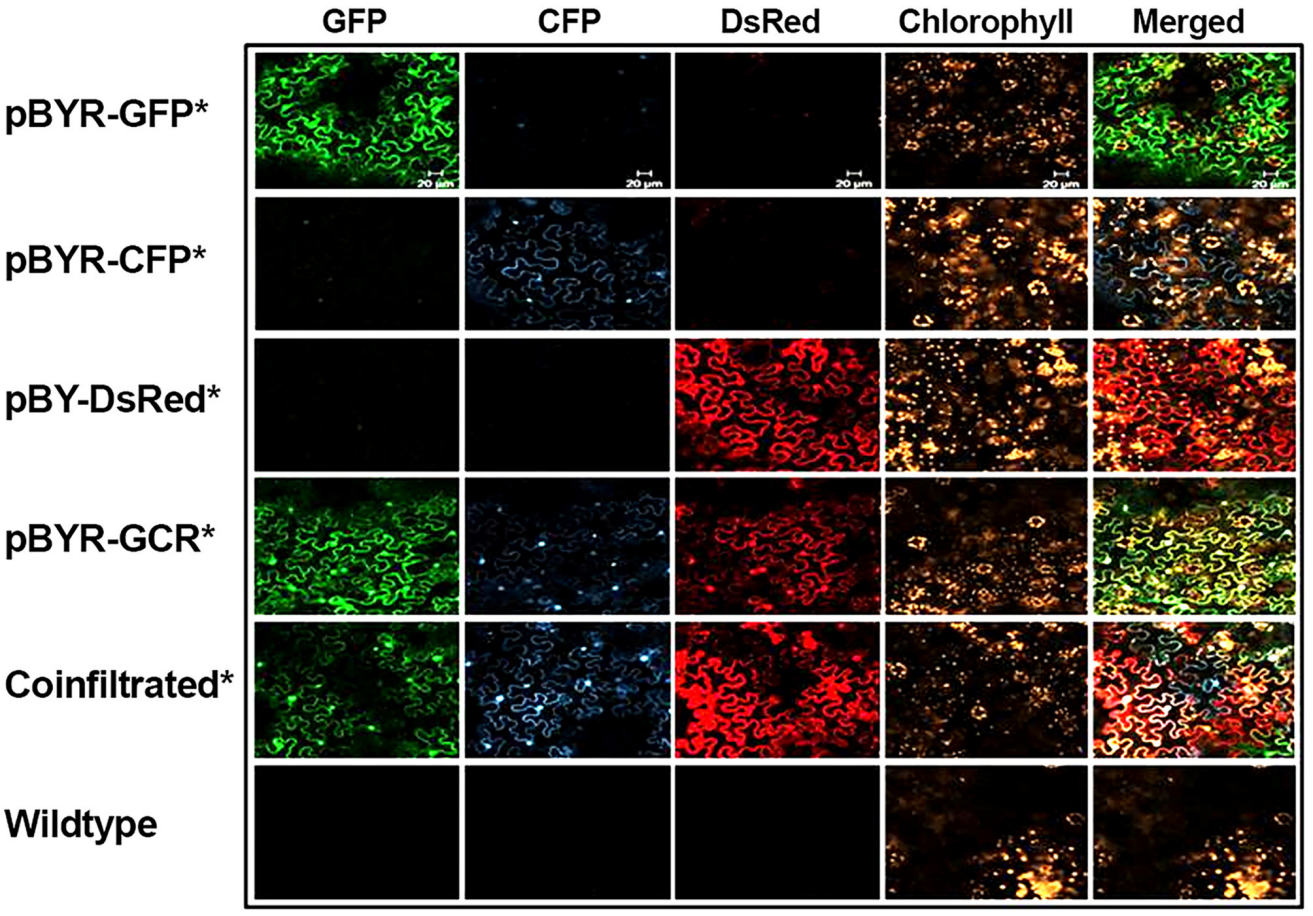
Simultaneous co-expression of three fluorescent proteins. *N. benthamiana* leaves were infiltrated with *Agrobacterium* strains harboring expression vectors as indicated (on the left). At 2 DPI the infiltrated leaf samples were examined with confocal laser scanning microscope. For co-infiltration, mixture of *Agrobacterium* strains harboring pBY-GFP, pBY-CFP, and pBY-DsRed were used. Excitation lasers of 488, 458, and 543 nm and detection windows of 550–560, 470–500, and 614–646 nm were employed to detect GFP, CFP, and DsRed signals, respectively. For plant autofluorescence chlorophyll detection, the excitation laser of 633 nm with detection window of 630–700 nm was used.

Next, to study the effects of expressing three proteins simultaneously, BeYDV replicon vectors were created with expression cassettes for GFP, DsRed, and CFP. The vector pBY-GCR (Figure 1) was designed to contain varied 5′ UTRs and 3′ UTRs to test whether unwanted recombination or RNA silencing might result from repeated genetic elements. Alternatively, individual expression cassettes for GFP, DsRed, and CFP which contained identical optimized vector components were coinfiltrated. By non-reducing SDS-PAGE, CFP runs slightly below GFP despite its similar monomeric size of ~26 kDa, allowing the fluorescence of each protein to be observed independently. The optimized vector produced a substantial increase of all three fluorescent proteins compared to the unoptimized vector, indicating that if there is a detrimental effect of repeated genetic elements, it is outweighed by the benefit gained from using optimized genetic elements (Figures 2B,C, compare “GCR old” to “GCR”). Producing three proteins simultaneously resulted in the same total yield of recombinant protein (Figure 2B, reducing lanes) (Figure 2B, lane “GCR”). An analysis of replicon formation revealed that the single large replicon displayed somewhat reduced replication, however high accumulation of replicons was still observed in all constructs (Figure 3C). Confocal microscopic examination revealed colocalized green, cyan, and red fluorescence, indicating efficient simultaneous expression of the three different proteins in each cell. In contrast, leaf samples individually infiltrated with GFP, DsRed, or CFP alone showed only a single fluorescent signal (Figure 4).

Finally, to test simultaneous expression of four proteins, an optimized YFP vector was coinfiltrated with optimized GFP, DsRed, and CFP vectors. The ~26 kDa YFP runs at a similar size to GFP on non-reducing SDS-PAGE but is yellowish/green in appearance under UV illumination. The sample containing all four proteins had slightly reduced DsRed/CFP fluorescence, but an increased green/yellow fluorescence, likely due to the combined GFP/YFP bands overlapping (Figure 2B, lane GCRY). Compared to co-expression of three proteins, the total recombinant protein yield was not significantly reduced when all four proteins were coexpressed (Figure 2B, reducing lanes and Figure 2C). These data suggest that optimized BeYDV vectors can produce high levels of up to 4 proteins simultaneously.

### Expression of mAbs Using Optimized Plant Expression Vectors

**Figure 5:**
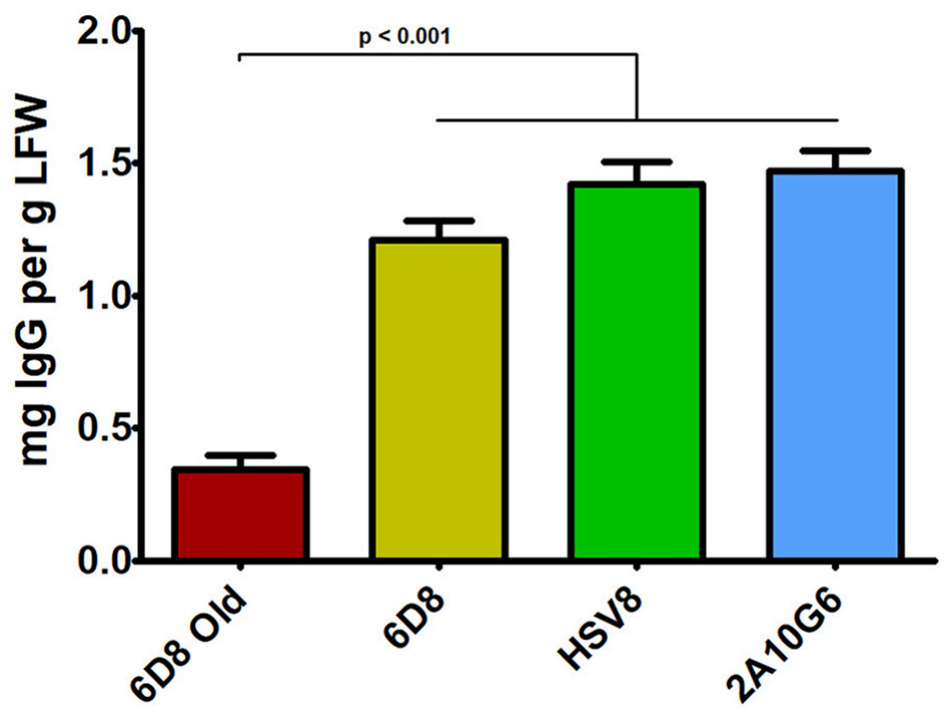
IgG production of three mAbs using optimized plant-expression vectors. Leaves of *N. benthamiana* agroinfiltrated with unoptimized (6D8 old) or optimized (6D8, HSV8, c2A10G6) BeYDV vectors were harvested at 5 DPI and protein extracts were analyzed for IgG production by ELISA using human IgG as a reference standard. Columns represent results from three independently infiltrated leaf samples ± standard error.

**Figure 6:**
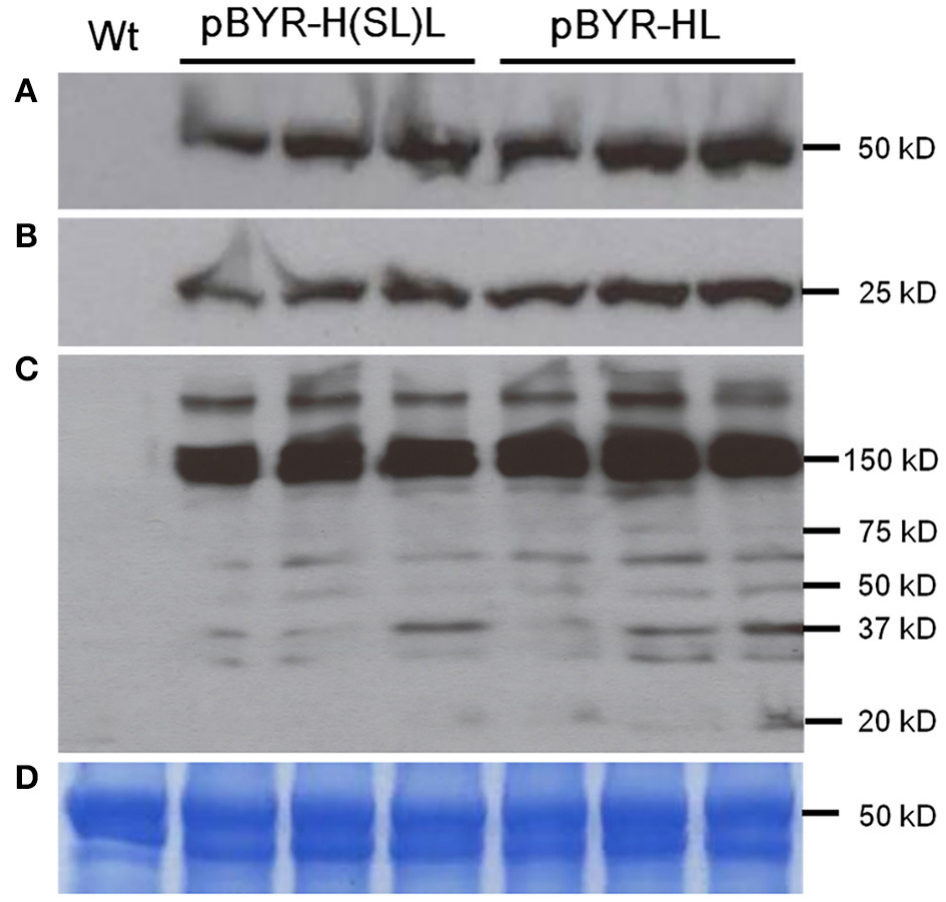
Western blot analysis of plant-derived 6D8. Protein samples were separated on a 4-20% SDS-PAGE gradient gel under denaturing and reducing condition **(A,B)** or under non-reducing condition **(C)** and blotted onto a PVDF membrane. The membrane was incubated with a goat anti-human gamma chain antibody or goat anti-human kappa chain antibody to detect heavy chain **(A)** or light chain **(B,C)**. Wt: Protein samples extracted from uninfiltrated leaves; lanes marked pBYR-H(SL)L, protein samples extracted from the leaves infiltrated with dual replicon construct pBYR-H(SL)L; lanes marked pBYR-HL, protein extracted from the single replicon construct pBYR-HL. **(D)** Commassie blue stained gel is shown for normalized total protein loading.

Three mAbs were chosen for expression using optimized BeYDV vectors. We have previously described production of the humanized 6D8 (6D8), an IgG1 targeting the Ebola virus glycoprotein GP1 (Huang et al., 2010). The antibody 2A10G6 recognizes an epitope in the highly conserved fusion loop on the flavivirus envelope protein, allowing it to effectively neutralize a wide range of flaviviruses. To create a more humanized form of 2A10G6, the variable regions in 6D8 were replaced with the variable regions from 2A10G6 (referred to as chimeric 2A10G6 or c2A10G6). Lastly, we also produced the mAb HSV8, a human-derived antibody which neutralizes herpes simplex virus (HSV) in mice and traps HSV in human cervicovaginal mucus (Zeitlin et al., 1996; De Logu et al., 1998; Schroeder et al., 2018). Optimized BeYDV vectors containing the plant codon-optimized c2A10G6, 6D8, and HSV8 heavy and light chain coding sequences were agroinfiltrated into glycoengineered *N. benthamiana* that produces highly homogenous mammalian-like glycans (Strasser et al., 2008; Castilho and Steinkellner, 2012; Montero-Morales and Steinkellner, 2018). Since the antibodies described in this study have potential as human therapeutics, it was important to use an expression host capable of creating antibodies that contain a mammalian-like glycosylation pattern (Marusic et al., 2018). To maintain consistency, only glycoengineered plants were used for the antibody expression experiments. Then, antibody production from the leaves were quantified using ELISA designed to detect fully assembled IgG. Using an unoptimized vector (Huang et al., 2010), 6D8 was produced at a level of 0.38 mg/g LFW while the optimized vectors produced 1.21 mg/g LFW 6D8, 1.42 mg/g LFW HSV8, and 1.47 mg/g LFW 2A10G6 (Figure 5). To determine whether replicon configuration affects IgG production, vectors containing the expression cassettes for the 6D8 heavy and light chains were either placed in a large single replicon (pBY-HL) or individual smaller replicons (pBY-H(SL)L). In agreement with our results using GFP and DsRed, no differences in expression were observed between the configurations, indicating the flexibility of BeYDV replicons in co-expressing multiple proteins (Figure 6).

### Characterization and Purification of Chimeric 2A10G6

**Figure 7:**
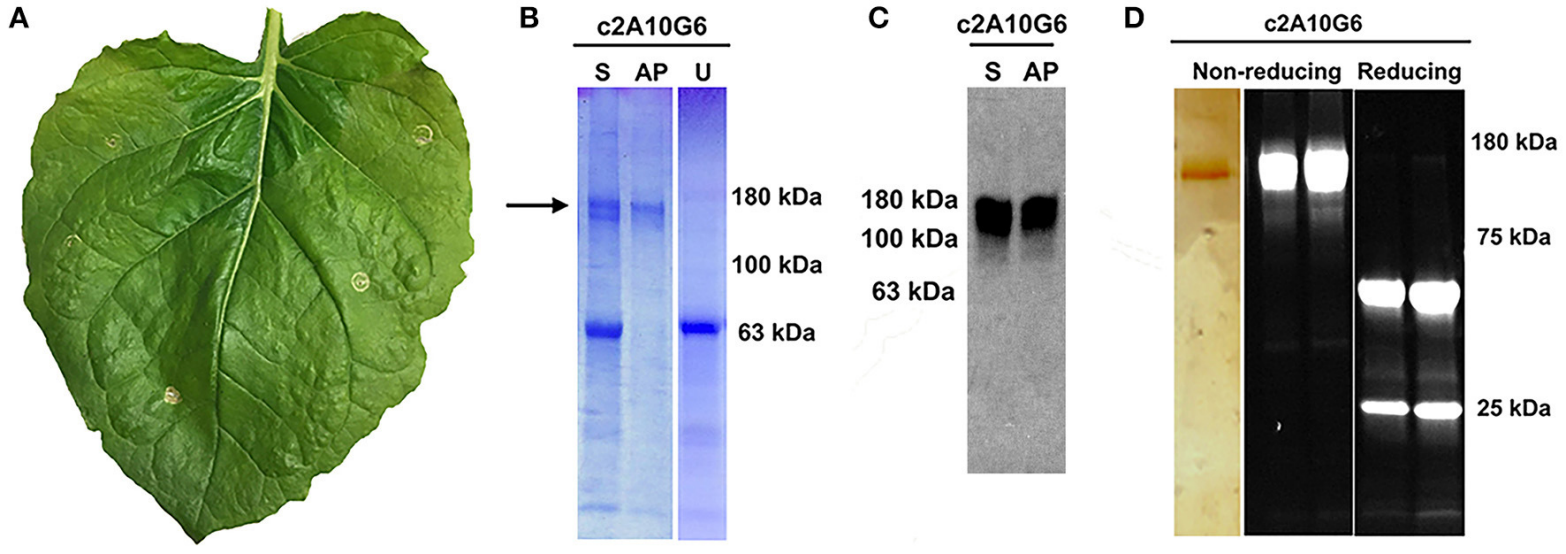
Characterization of c2A10G6. **(A)** A *N. benthamina* leaf at 4 DPI was examined for signs of chlorosis or necrosis. Faint chlorosis was visible, but there was no visible necrosis. **(B)** To test whether the clarified leaf extracts of the antibody constructs were stable upon acid-precipitation, 1N phosphoric acid was added to a final acid volume of 4% of the total soluble extract. Following a 6-min incubation, the samples were neutralized with 1M Tris base. For comparison, a sample of the leaf extract pre-acid precipitation was also included along with a control uninfiltrated leaf extract that was not treated with acid. All three samples were mixed with non-reducing sample buffer and loaded on a 4–15% polyacrylamide gel for analysis by Coomassie-staining. **(C)** The same samples described in part A were run on a 4–15% polyacrylamide gel for analysis by Western blot. The Western blot was detected with HRP-conjugated goat anti-human IgG (kappa only). **(D)** After protein G affinity purification, samples of the purified antibody were run on SDS-PAGE gels under non-reducing or reducing conditions as noted. The left panel shows the results following a silver stain. Only the c2A10G6 band is visible. The two panels on the right show the results of the purified antibody run under non-reducing and reducing conditions on a stain-free gel. S, soluble fraction pre-acid precipitation; AP, samples subjected to acid-precipitation; U, uninfiltrated clarified leaf extract; NR, non-reducing conditions; R, reducing conditions.

Optimized vectors contain components designed to reduce cell death (Diamos et al., 2016; Diamos and Mason, 2018c). As c2A10G6 had not been previously made in plants, leaves infiltrated with c2A10G6 were analyzed at 4 DPI for signs of chlorosis or necrosis which would suggest that the construct was toxic to the plant. However, there was no visible necrosis and faint chlorosis, indicating that antibody accumulation in the leaves was well tolerated (Figure 7A). To test whether the antibody was correctly assembled, clarified protein extracts from leaf samples were separated by SDS-PAGE. On both Coomassie-stained gel and western blot, a prominent band at the fully assembled heterotetrameric size of ~150 kDa was visible in the samples agroinfiltrated with c2A10G6 but not in the uninfiltrated control (Figures 7B,C). This further confirmed the high level of expression since the antibody band was prominent in a clarified plant extract, even before purification. The clarified leaf samples were then assessed by acid precipitation to verify if the antibody was stable after exposure to low pH conditions. Since acid precipitation removes plant contaminants that could otherwise hinder purification, it is a useful method to enrich leaf extract containing antibodies. Samples of clarified plant extract were briefly exposed to low pH conditions (~pH 4.1), then raised back to a neutral pH. Upon acid precipitation, several plant contaminant bands were removed, including the abundant rubisco large subunit band (~63 kDa), resulting in substantial enrichment of the antibody (Figure 7B). After purification by protein G affinity chromatography, samples assessed under non-reducing and reducing conditions on both a silver-stained gel and a stain-free gel show that the samples were highly pure (>95%) with very little degradation (Figure 7D).

### c2A10G6 Recognition of the Fusion Loop Epitope

**Figure 8:**
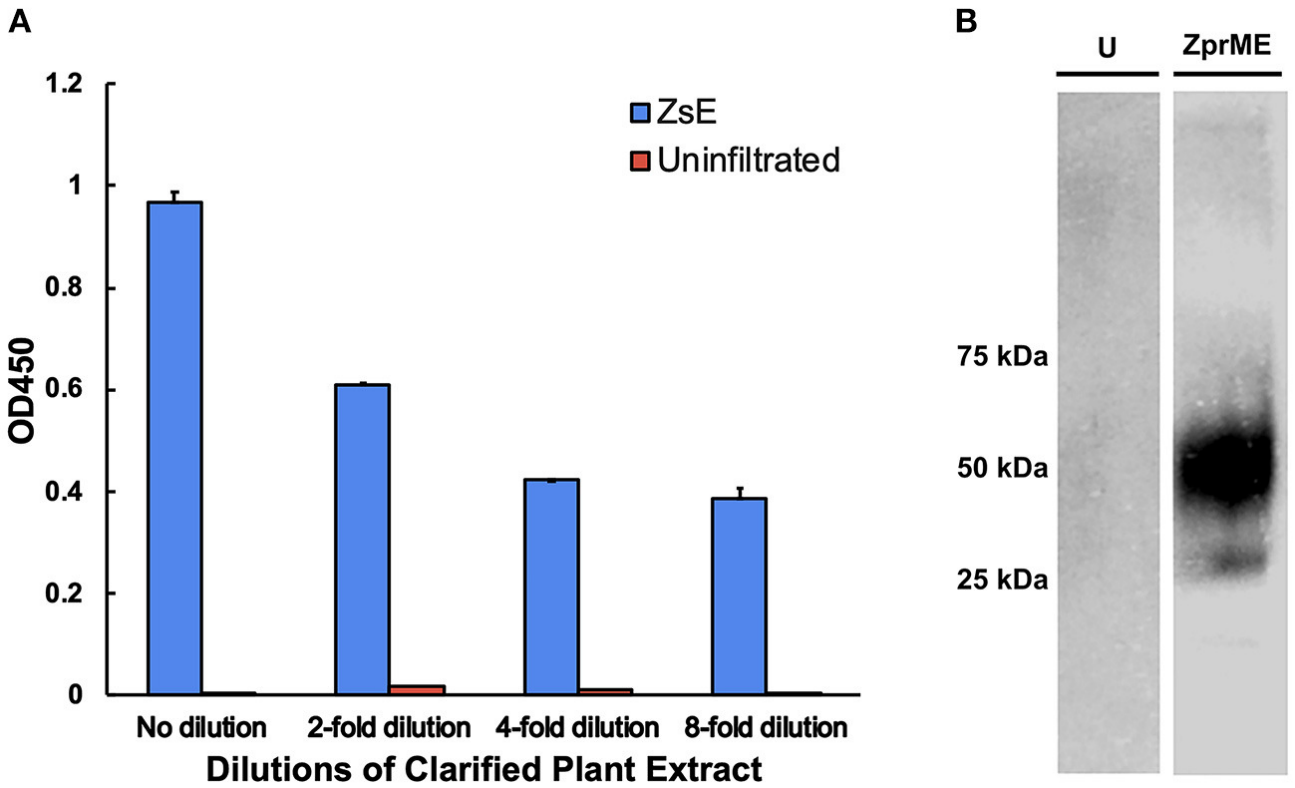
Binding of c2A10G6 to ZIKV envelope glycoprotein. **(A)** Varying dilutions of clarified protein extract containing ZsE was directly bound to a polystyrene plate and probed with purified c2A10G6 to assess whether the antibody recognized the fusion loop epitope. Bound antibody was detected with goat anti-human IgG (kappa only) HRP conjugate. An uninfiltrated negative control was included to assess the level of any non-specific binding to native plant proteins. **(B)** The ability of c2A10G6 to recognize the fusion loop epitope was analyzed via a western blot. Clarified protein extracts containing ZprME or an uninfiltrated control were probed with purified c2A10G6 and detected with HRP-conjugated goat anti-human IgG antibody.

Since c2A10G6 is a chimeric version of the murine mAb 2A10G6 that recognizes a highly conserved fusion loop on ZIKV, it was necessary to verify that the chimeric version retains the ability to recognize and bind the fusion loop epitope. First, the soluble ectodomain of the Zika envelope protein (ZsE, amino acids 1-403) that contains the fusion loop was expressed in plants. Next, a direct ELISA was conducted in which a clarified ZsE plant extract was directly bound to a 96 well high-binding polystyrene plate and probed with serial dilutions of purified c2A10G6. Uninfiltrated leaf extract was also included in the experiment to ensure there was no cross-reactivity with native plant proteins. As shown in Figure 8A, the chimeric antibody recognized the Zika soluble envelope while having negligible reactivity with native plant proteins. As an additional test, Zika prME was also expressed in plants and probed via western blot with c2A10G6. The antibody reacted with the prME-containing extract but not an uninfiltrated leaf extract (Figure 8B). Together, these data show that the chimeric antibody retains its ability to bind the Zika envelope protein.

### Neutralization Activity of c2A10G6 Against ZIKV

**Figure 9:**
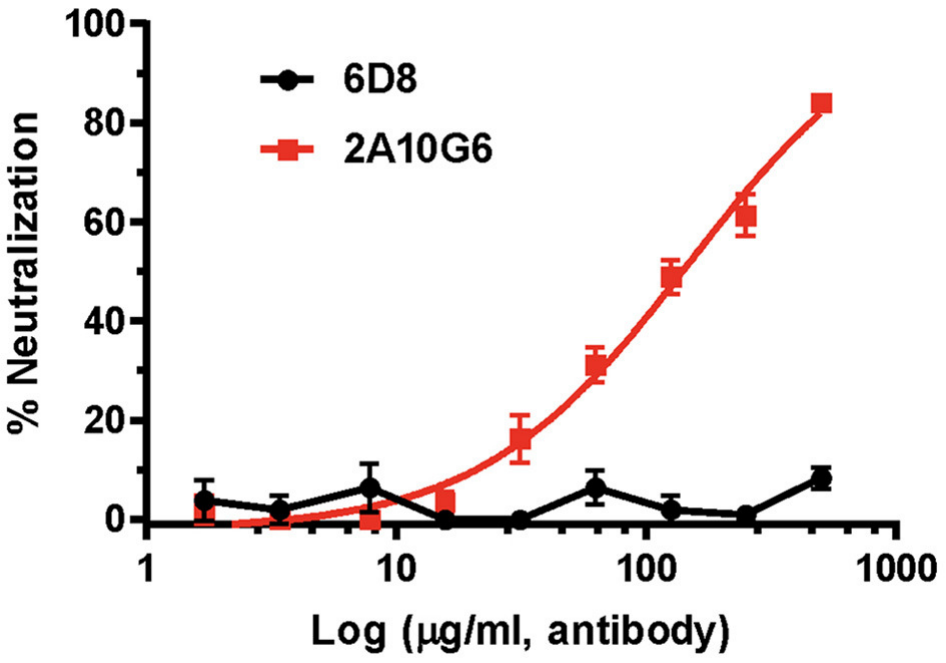
The neutralizing activity of c2A10G6 against ZIKV. Dilutions of c2A10G6 or 6D8 (negative control) were co-incubated with 100 PFU of ZIKV and Vero cells for 3 days. Plaques were counted and percent neutralization and EC_50_ were calculated. Experiments were performed twice with technical triplicates for each sample. Bars represent the standard deviation (SD) of the mean.

Plaque reduction neutralization test (PRNT) was used to evaluate the neutralization potential of plant produced mAbs against ZIKV (Yang et al., 2017). While the negative control 6D8 mAb (an anti-Ebola mAb) did not neutralize ZIKV, c2A10G6 showed strong neutralizing activity against ZIKV (Figure 9). The mean EC_50_ value of plant-derived chimeric c2A10G6 (EC_50_ = 148 μg/ml) is superior to that of hybridoma-produced mouse c2A10G6 (EC_50_ = 249 μg/ml) (Dai et al., 2016), indicating at least comparable if not better potency. Overall, the chimeric c2A10G6 produced in plants is fully functional and exhibited potent neutralizing activity against infectious ZIKV.

## Discussion

Plant expression systems have properties which make them uniquely suited to produce certain biopharmaceuticals. For example, when made in mammalian cells, the production of glucocerebrosidase as an enzyme replacement therapy for Gaucher's disease required costly *in vitro* glycosylation. However, as plants already contained the required glycoform, plant-based production of glucocerebrosidase reduced costs as well as potentially improved consistency and efficacy (Zimran et al., 2011). These advances led to the first FDA approval of a plant-based therapeutic (Fox, 2012) while further research has led to the development of a lyophilized carrot cell juice which restores healthy enzyme levels in patients when consumed orally (Shaaltiel et al., 2015). As another example of the unique potential of plant systems, an anti-cancer lectin that was found to be toxic to mammalian cells and that was not properly folded in *E. coli* could instead be efficiently produced in plants, with an estimated 80% reduction in costs and 3-fold improved potency (Gengenbach et al., 2019). Similarly, while human phosphatase and actin regulator 1 (PHACTR1) is difficult to express in mammalian cells due to a large number of interacting partners and cannot be properly folded in bacterial systems, it can be efficiently made in plants (Gengenbach et al., 2018). The advances in the field of plant-made antibodies can be clearly seen by the first in-human clinical trial of the anti-cancer antibody 2G12 made in transgenic tobacco plants (Ma et al., 2015). Recently, a plant-made quadrivalent influenza VLP vaccine showed sustained, strong cross-reactive immune responses and has advanced to phase 3 clinical trials (Pillet et al., 2016, 2019).

In many cases, co-expression of two or more proteins is required to produce biologically active pharmaceutical proteins, such as monoclonal antibodies, IgA, or multi-component virus like particles (Thuenemann et al., 2013; Palaci et al., 2019). To further improve the utility of plant-based systems, we have designed an expression system that is capable of efficiently producing heteromultimeric proteins (Huang et al., 2010). In this study, by optimizing the genetic elements of the vector, we were able to simultaneously coexpress two, three, or four proteins at up to 2.5-fold the expression level of our previous, unoptimized vectors (Figures 2, 5). We have identified many genetic components that can provide a wide range of expression levels in plant systems (Diamos and Mason, 2018a). While using multiple expression cassettes with identical genetic components results in similar production levels of each individual protein, using suboptimal genetic components for some expression cassettes would allow fine-tuning of the expression level of each individual protein subunit. This may be ideal for heteromultimeric proteins that require different ratios of each subunit, or in cases where multiple proteins need to be expressed at low levels (Diamos et al., 2019a). Optimized BeYDV vectors contain highly efficient double terminators which may inhibit induction of silencing signals by RDR6 (Luo and Chen, 2007; Baeg et al., 2017; Diamos and Mason, 2018a), as well as the P19 suppressor of RNA silencing. While RNA silencing mechanisms may cross-react with expression cassettes containing duplicated genetic elements (Zischewski et al., 2016), we observed no loss of total yield when two, three, or four expression cassettes containing identical 5′ and 3′ UTRs were coinfiltrated (Figure 2C). Furthermore, using varied but suboptimal 5′ and 3′ UTRs clearly negatively impacted expression (Figure 2C). Since no obvious detriment has yet been observed upon co-expression of additional proteins, we suspect it is also possible for these vectors to efficiently produce more than four proteins in the same cell due to the non-competitive nature of the BeYDV replicons.

BeYDV requires only two viral cis elements, the LIR and SIR, for replication via the Rep/RepA proteins (Jeske, 2009). This feature, along with the general high fidelity of DNA-based systems, facilitates insertion of large amounts of heterologous genetic information into a BeYDV vector. A notable finding from the current study is that BeYDV replicons can be enlarged as much as 280% of the native viral replicon size without notable detrimental effect on gene expression. Transient infiltration of constructs pBY-GR and pBY-GCR resulted in efficient formation of ~5.4 and ~7.1 kb replicons, respectively (Figure 3). Interestingly, while accumulation of the enlarged replicons was decreased by 38–64% compared to the smaller replicon, the vector pBY-GR resulted in GFP and DsRed expression that was comparable with pBY-G(SL)R (Figure 3B). We have previously found that recombinant BeYDV vectors produce more replicons than are needed to achieve maximal expression, and thus reduced replication may be neutral or even beneficial, as the accumulation of excess replicons can enhance cell death (Diamos and Mason, 2018c). Notably, none of the optimized constructs used in this study produced significant cell death by the optimum harvest date. The maximum amount of genetic information that can be placed into a BeYDV replicon vector without compromising the expression level remains to be studied. However, the need to rely on placing multiple expression cassettes into a single large replicon is circumvented by the fact that multiple smaller replicons can be delivered in the same T-DNA vector without any notable loss of efficiency (Figures 3, 6). Together, these results highlight the flexibility of the BeYDV system, which can efficiently coexpress multiple proteins in a variety of configurations: e.g., by coinfiltrating multiple T-DNA vectors, by linking multiple individual replicons released from a single T-DNA by the nicking and ligating properties of Rep/RepA, or by arranging multiple expression cassettes in tandem inside of a single large replicon.

In recent years, monoclonal antibodies have been widely explored for use in immunotherapies and treatments for infectious diseases. One active area of research in antibody development focuses on the production of therapeutic mAbs against flaviviruses. The *Flaviviridae* family contains over 53 viruses, including many major health pathogens such as Dengue virus, yellow fever virus, West Nile virus, Japanese encephalitis virus, and ZIKV which create a significant global public health burden (Sun et al., 2018). For Dengue alone, it is estimated that there are over 390 million cases annually. An ideal mAb therapeutic against flaviviruses would: 1) be able to recognize and effectively neutralize multiple types of viruses; 2) be produced in an economical and cost-effective manner; and 3) be able to be easily purified (Deng et al., 2011). The antibody 2A10G6 recognizes an epitope in the highly conserved fusion loop found on the flavivirus envelope protein, allowing it to effectively neutralize infection of all four Dengue serotypes, yellow fever, and West Nile virus (Deng et al., 2011). 2A10G6 has also been shown to bind to and neutralize the Zika E protein (Dai et al., 2016). However, since the antibody is murine, direct delivery of the antibody to humans might result in an unwanted immune response to the antibody and result in swift clearance from circulation. One way of circumventing this problem is to create a humanized, chimeric version of the antibody in which the murine heavy and light chains are genetically fused into a human antibody. This type of antibody engineering has been studied since the 80s with great success leading to many marketed mAb treatments (Grilo and Mantalaris, 2019). Therefore, we designed a more humanized 2A10G6 antibody for expression in *N. benthamiana*. The chimeric antibody reached high levels of expression that exceeded the level that is considered to be commercially viable for plant-made antibodies (Nandi et al., 2016). Furthermore, the antibody was correctly assembled, purified to >95% homogeneity by a simple one-step purification process, and retained its ability to bind to Zika envelope protein with potent neutralizing activity (Figures 7B,C, 8A, 9).

To further demonstrate the general effectiveness of the BeYDV plant expression system, two other mAbs were produced. The anti-Ebola GP1 mAb 6D8 was produced using an unoptimized BeYDV vector (Huang et al., 2010) and thus served as a benchmark to compare updated vector configurations for mAb production. New optimized vectors (Figure 1) provided an approximate 4–5-fold increase in yield compared to the old unoptimized vectors (Figure 5), allowing milligram quantities of mAb to be produced from a single plant leaf. While previous vector iterations caused plant cell toxicity (Diamos and Mason, 2018c), no cell death was observed for c2A10G6 (Figure 8A) or any of the other mAbs used in this study (data not shown). Though this mAb yield is high, the relatively higher expression of the fluorescent proteins indicates that there are still inefficiencies for mAb production. Protein engineering has been used to remove certain motifs in mAb structure that are susceptible to degradation or instability in plants, allowing mAb production as high as 2 g/kg LFW (Zischewski et al., 2016). By engineering an optimal human IgG1 backbone for expression in plants, we anticipate further improvements could be made to this mAb production system.

## Data Availability Statement

The datasets generated for this study are available on request to the corresponding author.

## Author Contributions

AD, MP, SR, HS, QC, and HM designed experiments and analyzed data. AD, MP, SR, and HM constructed vectors. JH, BF, SR, and AD conducted fluorescent protein expression experiments. MP and AD conducted the antibody expression experiments. MP and MD conducted the antibody binding experiments. HS performed neutralization experiments. AD, JH, QC, and MP wrote the manuscript. AD and HM critically revised the manuscript.

### Conflict of Interest

The authors declare that the research was conducted in the absence of any commercial or financial relationships that could be construed as a potential conflict of interest.

## Abbreviations

BeYDV: bean yellow dwarf virus
mAb: monoclonal antibody
ZIKV: Zika virus
GFP: green fluorescent protein
YFP: yellow fluorescent protein
CFP: cyan fluorescent protein
TSP: total soluble protein
g/kg LFW: grams recombinant protein per kilogram leaf tissue fresh weight.
